# Spherical Attapulgite/Silica Aerogels Fabricated via Different Drying Methods with Excellent Adsorption Performance

**DOI:** 10.3390/ma16062292

**Published:** 2023-03-13

**Authors:** Zhixiang Zhu, Shengyuan Wang, Ya Zhong, Qi You, Jun Gao, Sheng Cui, Xiaodong Shen

**Affiliations:** 1 State Key Laboratory of Materials-Oriented Chemical Engineering, College of Materials Science and Engineering, Nanjing Tech University, Nanjing 210009, China; 2 Jiangsu Collaborative Innovation Center for Advanced Inorganic Function Composites, Nanjing Tech University, Nanjing 210009, China; 3 Suqian Advanced Materials Industry Technology Innovation Center, Nanjing Tech University, Suqian 223800, China

**Keywords:** spherical ATP/SiO_2_ aerogels, drying techniques, heat treatment, adsorption performance

## Abstract

Dye wastewater has caused great harm to the environment, which is an urgent problem to be solved. As typical three-dimensional porous materials, aerogels have attracted great interest in dye wastewater treatment. In this work, spherical attapulgite/silica (ATP/SiO_2_) gels were initially prepared by easily scalable sol-gel dripping methods and then dried to aerogels with three drying techniques, namely, supercritical CO_2_ drying (SCD), freeze-drying (FD), and ambient pressure drying (APD). The effect of the drying techniques and heat-treated temperature on the physical characteristic, morphological properties, microstructure, and chemical structure of the spherical ATP/SiO_2_ aerogels were investigated. The macroscopic morphology of the spherical ATP/SiO_2_ aerogels was homogeneous and integrated without local cracking. The average pore diameter and specific surface area of the spherical ATP/SiO_2_ aerogels prepared by the three drying techniques were in the range of 6.8–8.6 nm and 218.5–267.4 m^2^/g, respectively. The heat treatment temperature had a significant effect on the pore structure and the wetting properties of the aerogels. The 600 °C heat-treated aerogels were subjected to adsorption tests in methylene blue (MB) solution (60 mg/g, 100 mL), which exhibited a great adsorption capacity of 102.50 mg/g. Therefore, the resulting spherical ATP/SiO_2_ aerogels possessed multipath preparation and exhibited an efficient adsorption performance, with the potential to be applied as an adsorbent for dye wastewater.

## 1. Introduction

Highly toxic, carcinogenic, and teratogenic dye wastewater has caused serious damage to the ecological environment [1,2]. Dyes in wastewater can destroy the self-purifying function of water bodies, since they can reduce water clarity, consume oxygen in the water, and hinder photosynthesis in plants [3]. Remarkably, most organic dyes are difficult to degrade naturally and can persist in the natural environment [4]. Methyl blue (MB) is a hydrophilic organic compound with free solubility in water, representing typical cationic dyes [5,6]. Adsorption has been recognized as one of the most efficient methods for removing dyes from wastewater due to the fact of its widespread use, effectiveness, and reusability. As typical three-dimensional porous materials, SiO_2_ aerogels have attracted great interest in adsorption fields [7]. SiO_2_ aerogels can be employed as dye adsorbents owing to the following two advantages: a high specific surface area that enables numerous adsorption sites and properties that can be easily tuned by surface modification to adapt to different application environments [8]. Yao et al. [8] used water as a solvent for preparing MTMS/tetraethoxysilane silica aerogels with a high porosity of 96.36%. Jiang et al. [9] synthesized amine-grafted silica aerogels by sodium silicate to adsorb CO_2_.

A SiO_2_ aerogel adsorbent can be obtained by solvent substitution and surface modification via the APD method. Regrettably, the hydrophobicity is unfavorable to the dispersion of aerogel in water and limits the adsorption of soluble dye. Previous research has suggested that heat treatment is a facile and high-efficiency method for converting hydrophobic SiO_2_ aerogels into hydrophilic ones. For instance, Yi et al. [10] compared the adsorption performance of hydrophobic and hydrophilic SiO_2_ aerogels on MB. The adsorption capacity of the hydrophilic SiO_2_ aerogel obtained by heat treatment at 500 °C was 9.53 mg/g, significantly higher than that of the hydrophobic sample (3.05 mg/g). Wei et al. [11] investigated the adsorption property of hydrophilic SiO_2_ aerogels obtained by calcinating hydrophobic aerogels at 600 °C for 3 h, which exhibited good adsorption performance for MB (51.16 mg/g). Some researchers also proved that metal ion solution modification is beneficial for increasing the adsorption capacity of aerogels on MB. Yang et al. [12] provided SiO_2_ aerogels modified with Mg^2+^ solutions, which displayed an adsorption capacity of 40.40 mg/g for MB. However, SiO_2_ aerogel materials are known for their poor mechanical behavior, which limits the practical applications of aerogels and is attributed to their fragility and brittleness. Hence, enhancing the mechanical properties without sacrificing its adsorption performance is a factor worthy of attention. One way is to optimize the micro/macro morphology of the aerogel through preparation [13]. Another method is to add materials to form an enhanced composite structure [14]. Additionally, it has been demonstrated that spherical aerogels have good fluidity, uniform stress, and strain, which can improve the elastic–plastic deformability and mechanical properties [15,16]. Fiber reinforcement is one of the most effective strategies for improving the mechanical property of aerogel materials. Tian et al. [17] synthetized glass fiber enhanced SiO_2_ aerogel, which exhibited a high flexural modulus (2.65 MPa). Slosarczyk [18] provided a synthesis method for a carbon fiber-SiO_2_ aerogel possessing an excellent elastic–plastic deformation ability. However, an obvious defect is that the structures of the reinforcement material and the aerogel are usually different, which will lead to huge stress in the composite material during the preparation and drying process [19]. To maintain the three-dimensional skeleton structure of an aerogel, plentiful and nanoscale natural clay with a unique structure and function is usually used to strengthen silicon-based aerogels, such as attapulgite [20], montmorillonite [21], and sepiolite [22].

Attapulgite (ATP) is a naturally available 1D hydrophilic clay, which displays the hydrated magnesium aluminum silicate structure with a theoretical composition of Mg(Al)_5_Si_8_O_20_(OH)_2_(OH_2_)_4_•4H_2_O [23]. The diameter of ATP is approximately 30 nm, and the length is between 0.5 and 1 µm, which is very close to the pore size of SiO_2_ aerogels (5–100 nm) [24]. In addition, ATP exhibits a 2:1 type chain laminar structure with plentiful -OH groups, which has good chemical compatibility with the chemical groups of SiO_2_ aerogels. Because of the porous internal channels, large specific surface area, and certain ion exchange capability, club-shaped ATP is also extensively applied as an adsorbent material [25,26]. Chen et al. [27] studied the removal rate and adsorption mechanism of calcined ATP for MB. With the increasing temperature, the adsorption capacity of ATP reached a maximum of 700 °C (78.11 mg/g). All of the above characteristics of ATP are beneficial to the preparation of high-performance ATP/SiO_2_ composite aerogel adsorbent. Zhang et al. [28] synthesized ATP/SiO_2_ aerogels, which displayed an adsorption quantity of 5.00 g/g for total petroleum hydrocarbon. Meanwhile, the structures and properties of ATP/SiO_2_ composite aerogels dried under air conditions were also investigated. Notably, drying technology is an important process for the synthesis of aerogels with a specific structure. The microstructures and properties of the final aerogels mainly depend on the drying technique. Hence, it is necessary to study the influence of different drying processes on the structure of ATP/SiO_2_ composite aerogels.

Currently, aerogels are usually obtained by three drying techniques, such as supercritical CO_2_ drying (SCD), freeze-drying (FD), and ambient pressure drying (APD) [29]. The SCD technique is an alternative drying process that avoids capillary pressure by replacing the solvent with supercritical fluid CO_2_. It is an ideal method to obtain aerogels with an intact structure and low density. The main disadvantages of the SCD method are the required energy, maintenance cost, and high-pressure conditions [30]. FD is the process of removing water or other solvents from frozen samples through sublimation in a vacuum environment. The freezing process can evade the shrinkage of the aerogel skeleton, but the crystallization of the solvent will enlarge the pore size and destroy the original pore structure [31]. The principle of APD is to elevate the temperature of the solvent above the boiling point and transform it into gas, which is the most convenient technique for removing solvents from porous materials. The convenient evaporation of the APD method with its low cost is very suitable for mass production and wide application [19,32]. However, the capillary pressure during the drying process exceeds the elastic limit of the solid structure, which may result in the shrinkage and collapse of the porous aerogel materials [33]. To reduce the effect of capillary forces and control the irreversible shrinkage of SiO_2_ aerogels during the APD process, surface hydrophobic modification by silylation is of great importance. Selay Sert et al. [34,35] investigated the effects of different silylation agents (including MTMS, trimethylchlorosilane, and methyltriethoxysilane) on the microstructure and chemical properties of SiO_2_ aerogels under APD. The results show that the MTMS-modified SiO_2_ aerogel had a highly developed three-dimensional network structure and high specific surface area. Therefore, MTMS was used as a silane modifier in this work.

To synthesize an excellent dye adsorbent, ecofriendly spherical ATP/SiO_2_ aerogels were successfully prepared via three drying methods (SCD, FD, and APD). Hydrophobization of the spherical ATP/SiO_2_ aerogels was achieved by MTMS modification. The structure of the as-prepared spherical ATP/SiO_2_ aerogels with three drying techniques was analyzed and compared. The effects of the heat treatment temperatures on the physicochemical properties of the resulting aerogels were studied. Furthermore, the adsorption performance of the spherical ATP/SiO_2_ aerogels dried by APD and heat treated at 600 °C was also investigated. The spherical ATP/SiO_2_ aerogels have excellent adsorption properties and can be prepared in multiple ways. This suggests that the prepared aerogels have promise as an adsorbent for dye wastewater.

## 2. Experimental Section

### 2.1. Materials

Attapulgite (ATP) was purchased from Jiangsu Jiuchuan Nanotechnology Co., Ltd (Xuyi, China). Alkaline silica sols (30 wt%) were obtained from Shanghai Yuanye Biological Technology Co., Ltd. (Shanghai, China). Dimethicone oil (1000 ± 80 mPa.s), acetic acid, and methylene blue (MB) were purchased from Shanghai Macklin Biochemical Co., Ltd. (Shanghai, China). Trimethoxymethylsilane (MTMS) and tert-butanol were supplied by Shanghai Aladdin Biochemical Technology Co., Ltd. (Shanghai, China). N-hexane was provided by Shanghai Lingfeng Chemical Reagent Co., Ltd. (Shanghai, China). Ethanol and deionized water (H_2_O) were supplied by Wuxi City Yasheng Chemical Co., Ltd. (Wuxi, China). The reagents used in the experiment were all analytically pure, without further purification.

### 2.2. Methods

#### 2.2.1. Synthesis of Spherical ATP/SiO_2_ Gels

The ATP/SiO_2_ solution was prepared according to the following steps. Alkaline silica sols, H_2_O, and ATP were directly separated in a pot with a mass ratio of 1:1:0.5. Acetic acid was used as a catalyst to adjust the pH value of the solution to 4.5~5.5. Then, the mixture was stirred for approximately 30 min at room temperature. Subsequently, the ATP/SiO_2_ solution was obtained by ultrasonic treatment for 15 min. Furthermore, the spherical ATP/SiO_2_ gels were prepared by the dripping method. The ATP/SiO_2_ solution was dripped into dimethicone oil at 80 °C to form spherical ATP/SiO_2_ gels. Finally, the spherical ATP/SiO_2_ gels were washed five times with deionized water at 60 °C to remove impurities and then aged in water for 12 h.

#### 2.2.2. Preparation of Spherical ATP/SiO_2_ Aerogels Using Different Treatment Techniques

The hydrophobic spherical ATP/SiO_2_ gels were obtained by the surface modification process. Trimethoxymethylsilane (MTMS) was employed as a hydrophobic modification agent. The spherical ATP/SiO_2_ gels were firstly placed in a mixed MTMS/ethanol solution with a volume ratio of 1:4 for 12 h and then soaked in ethanol solution for 24 h to wash off the excess MTMS and reaction byproducts. Subsequently, the primed samples were dried to hydrophobic spherical ATP/SiO_2_ aerogels using the SCD method in an autoclave for 4 h (50 °C, 10 MPa, HELIX 1.1 system, Applied Separations, Inc., Allentown, PA, USA), denoted as HAS-S. The samples without hydrophobic modification were denoted as AS-S. 

In addition, the spherical ATP/SiO_2_ gels were initially placed in a mixed MTMS/tert-butanol solution with a volume ratio of 1:4 for 12 h and then washed with tert-butanol solution for 24 h to exchange the excess MTMS and reaction byproducts. Finally, the hydrophobic spherical ATP/SiO_2_ aerogels were prepared using the FD method (frozen at −15 °C in a fridge for 5 h and then dried at −80 °C for 12 h), denoted as HAS-F.

Moreover, the previous hydrophobic spherical ATP/SiO_2_ gels were adequately washed with N-hexane for 24 h to displace the water and byproducts from the samples. The volume of N-hexane was 5 times larger than the volume of the gel. After aging and solvent exchange, the hydrophobic spherical ATP/SiO_2_ aerogels were obtained by APD in an air oven (50 °C for 6 h, and 80 °C for 4 h), denoted as HAS-A. 

To study the variation in the physicochemical properties of the spherical aerogels, the HAS-A was heat treated at different temperatures (200 °C, 400 °C, 600 °C, and 800 °C) for 3 h in a muffle furnace with a heating rate of 5 °C/min. The 600 °C heat-treated HAS-A is denoted as AS-A.

### 2.3. Characterization

The apparent densities (ρ) of the samples were calculated by ρ = m/v, where m and v are the apparent mass and volume. Scanning electron microscope (SEM) images of the samples were carried out using an Ultra-55 (Zeiss, Oberkochen, Germany) at an operating voltage of 15.0 kV. Transmission electron microscope (TEM) images were recorded on a JEOL JEM 2100F microscope at 200 kV. X-ray photoelectron spectroscopy (XPS) was carried out using an Axis Ultra DLD equipped with Al Ka (1486.6 EV). Fourier transform infrared spectroscopy (FT-IR) measurements were recorded on an FT-IR spectrometer (Spectrum 100, Perkin Elmer, Waltham, MA, USA) for KBr pellets. The contact angles were carried out using a JC2000D1 by grinding the aerogels into powder and then pressing them into pieces. The thermogravimetric (TG) analysis was conducted on a Netzsch STA449F5 thermal analyzer (Selb, Germany) in a temperature range of 30 °C to 800 °C with a heating rant of 10 °C/min under flowing air. The BET specific surface area (S_BET_), pore volume, and pore distribution were measured using a V-sorb 2800P after pretreatment for 6 h at 120 °C. The zeta potential was analyzed and measured using a Malvern Zetasizer Nano ZS90 (Malvern, UK).

### 2.4. Adsorption Experiment

The AS-A was used as an adsorbent for studying the adsorption performance. The MB solution was used to simulate the wastewater, and all adsorption tests were carried out at room temperature. A total of 50 mg AS-A and 100 mL MB solution were mixed in a centrifuge tube and shaken for 100 min (200 rpm/min) at room temperature. After a sufficient adsorption process, the solution was centrifuged for 5 min (5000 r/min), and the concentration of residual dye in the supernatant was analyzed using a UV-Vis spectrometer at 663 nm. The influence of the pH, contact time, and MB concentration were systematically studied to determine the adsorption performance of the AS-A for MB. The removal rate of MB was calculated by a comparison of the initial concentration and the residual concentration after adsorption. The equations for the adsorption efficiency (*R*) and the adsorption capacity (*q_e_*) of AS-A are shown as follows:

(1) $$\;R=\frac{C_{0}-C_{e}}{C_{0}}\times 100\%$$

(2) $$\;q_{e}=\frac{(C_{0}-C_{e})V}{m}$$

where *C*_0_ and *C_e_* represent the initial and residual concentration of MB (mg/L), respectively; *V* is the volume of the MB solution (L); and m is the mass of AS-A (g).

## 3. Results and Discussion

### 3.1. Formation Mechanism of the Spherical ATP/SiO_2_ Aerogels

**Figure 1:**
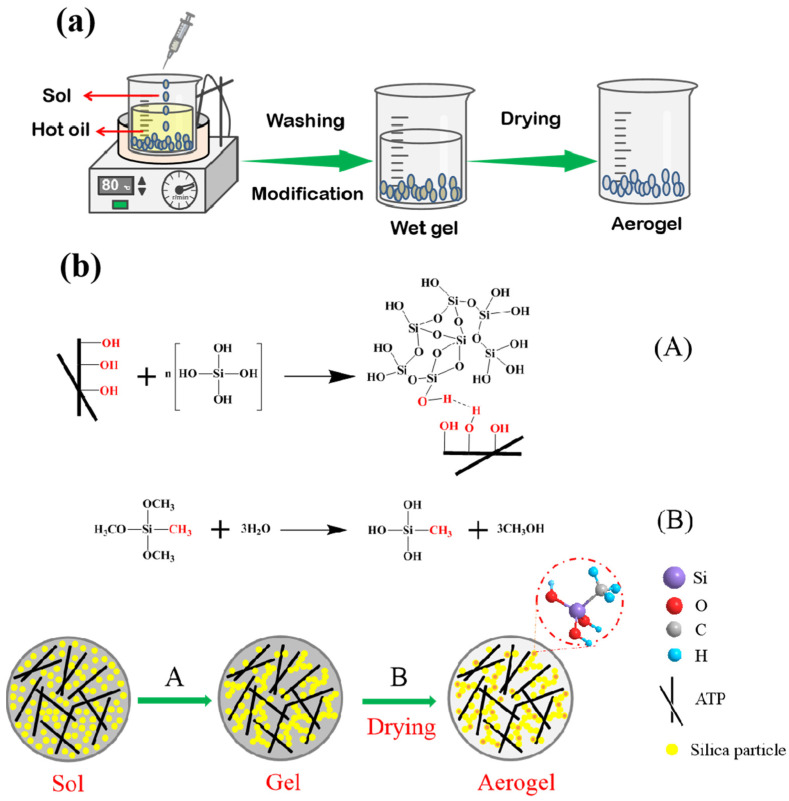
A schematic diagram of the (**a**) synthesis process and (**b**) synthesis mechanism for the spherical hydrophobic ATP/SiO_2_ aerogels.

The schematic diagram of the synthesis process for hydrophobic ATP/SiO_2_ aerogels is shown in Figure 1a. Because of the incompatibility between water and oily solvent, the hydrophilic ATP/SiO_2_ mixed sols can disperse in the oily solvent. When the surface tension of the sol droplet is much greater than the gravity, the isotropic spherical ATP/SiO_2_ sol forms in the oil phase [16]. Considering the influence of the temperature on sol-gel kinetics, the oil bath temperature was set at 80 °C for forming the spherical ATP/SiO_2_ gel [9]. The final spherical ATP/SiO_2_ aerogels were obtained by three drying methods. Figure 1b presents the synthesis mechanism of the spherical hydrophobic ATP/SiO_2_ aerogels. Silica sols exist as nanoscale silica particles, which are distributed uniformly in the water. Both silica sol particles and ATP contain many active -OH groups, which can favorably translate into the ATP/SiO_2_ gels by a hydrolysis polycondensation reaction under the appropriate conditions of the silica concentration, pH value, and temperature. In addition, the silica sol is bound with ATP via hydrogen bonding, and then the MTMS used as hydrophobic modifier are grafted onto the ATP/SiO_2_ gels by the immersion modification method. 

### 3.2. Effect of Drying Techniques and MTMS Modification on Physical Characteristic and Morphological Properties of Spherical ATP/SiO_2_ Aerogels

**Figure 2:**
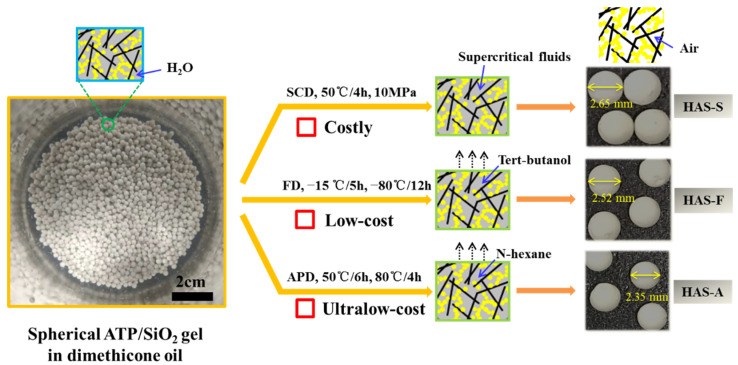
Comparison of the different drying methods for preparing the spherical ATP/SiO_2_ aerogels.

A comparison of the different drying methods for preparing the spherical ATP/SiO_2_ aerogels is shown in Figure 2. All spherical ATP/SiO_2_ aerogels exhibited an integrated macroscopic morphology with a diameter of approximately 2.5 ± 0.15 mm (2.65 mm, 2.52 mm, and 2.35 mm for HAS-S, HAS-F, and HAS-A, respectively). By contrast, the shrinkage rate of the HAS-A was larger than the other samples resulting from the drying capillary pressure of the solvent evaporation during the APD process. Table 1 shows the physical parameters of the ATP and spherical ATP/SiO_2_ aerogels prepared by different drying methods. The densities of the ATP/SiO_2_ aerogel composites (0.46–0.65 g/cm^3^) decreased greatly due to the loose three-dimensional skeleton structure instead of the relatively dense structures of the ATP (2.41 g/cm^3^). Meanwhile, the densities of the ATP/SiO_2_ aerogels modified by MTMS were larger than the samples without hydrophobic modification, which is attributable to the grafting of CH_3_-(SiOH)_3_ groups.

**Figure 3:**
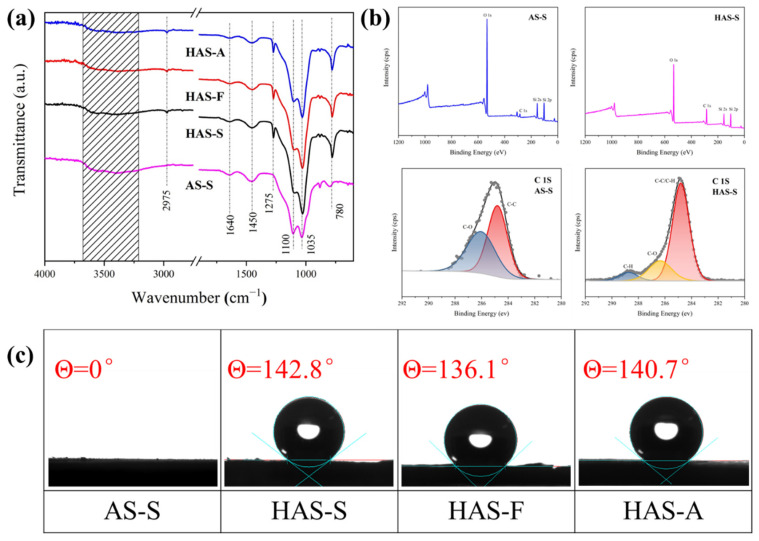
(**a**) The FT-IR spectra of AS-S, HAS-S, HAS-F, and HAS-A; (**b**) XPS spectra and C 1s narrow-scan spectra of AS-S and HAS-S; (**c**) water contact angles of AS-S, HAS-S, HAS-F, and HAS-A.

The FT-IR spectra of the as-prepared aerogels are presented in Figure 3a. A broad transmittance appeared at approximately 3200–3700 cm^−1^, which is attributed to the stretching vibrations of -OH groups [36]. The symmetric stretching and tetrahedra bending vibration of Si-O-Si and Si (or Al)-O appeared at 1035 cm^−1^ and 1100 cm^−1^ [37]. The peaks at approximately 1450 cm^−1^ and 1640 cm^−1^ are assigned to the deformation vibration of the adsorbed water and bound water [32,38]. By contrast, there was a new peak at 780 cm^−1^, which is normally observed in the hydrophobic group and corresponds to the stretching vibration of -CH_3_ in MTMS [39]. Moreover, all of the modified samples showed the symmetric deformation vibration of Si-C bonds and symmetric stretching vibrations of the terminal -CH_3_ groups at 2975 cm^−1^ and 1275 cm^−1^,which is proof of the presence of Si-CH_3_ [40]. Thus, the FT-IR spectrums have proved that the hydrophobic -CH_3_ groups were successfully coupled to the HAS-S, HAS-F, and HAS-A. The XPS measurement (Figure 3b) was subsequently used to quantify the elemental composition of AS-S and HAS-S. The XPS spectra of AS-S and HAS-S show four peaks at 100, 150, 285, and 531 ev, which are assigned to Si 2s, Si 2p, C 1s, and O 1s, respectively [39]. Compared with AS-S, the relative strength of the C 1s peak of the HAS-S increased significantly, while that of the O 1s peak became weak. It can be inferred that with the addition of MTMS, the dehydration condensation reaction between the -OH and CH_3_-(SiOH)_3_ groups resulted in the loss of some O elements and the grafting of -CH_3_ groups. The surface wettability of the spherical ATP/SiO_2_ aerogels was evaluated by contact angle measurements, which are presented in Figure 3. Due to the large number of –OH, the unmodified AS-S exhibited obvious hydrophilicity with the water contact angle values of 0°. Due to the existence of numerous -CH_3_ groups, the spherical ATP/SiO_2_ aerogels obtained by MTMS modification possessed excellent hydrophobic performance, corresponding to the values of 142.8°, 136.1°, and 140.7° for HAS-S, HAS-F, and HAS-A, respectively.

**Figure 4:**
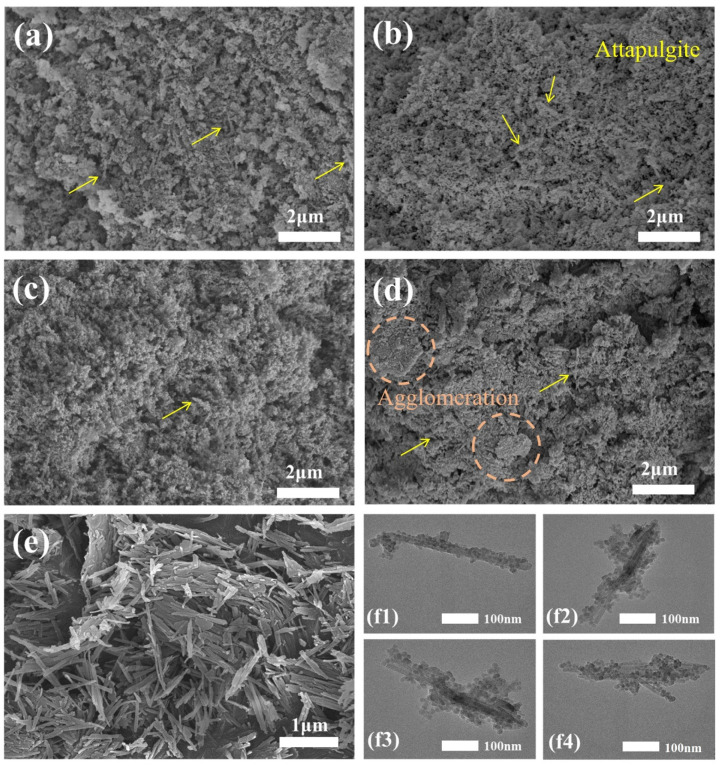
SEM images: (**a**) AS-S; (**b**) HAS-S; (**c**) HAS-F; (**d**) HAS-A; (**e**) ATP. TEM images: (**f1**) AS-S; (**f2**) HAS-S; (**f3**) HAS-F; (**f4**) HAS-A.

**Figure 5:**
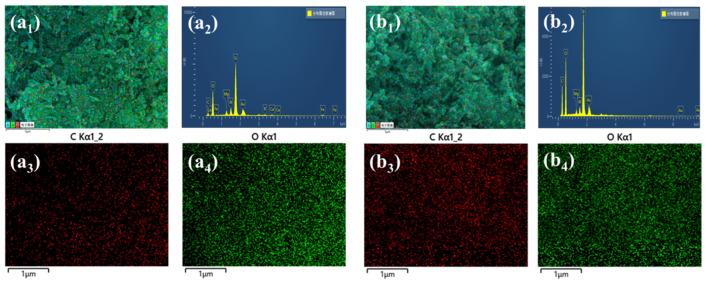
The EDS spectrum of (**a_1_**–**a_4_**) AS-S and (**b_1_**–**b_4_**) HAS-S.

Figure 4 shows the SEM and TEM images of the prepared spherical ATP/SiO_2_ aerogels and pure ATP. As shown in Figure 4e, the pure ATP displays the features of a club-shaped structure with a diameter of approximately 30 nm and partial agglomeration. As shown in Figure 4a–d, all of the ATP/SiO_2_ aerogels exhibited inherent three-dimensional porous network structures, which consisted of club-shaped ATP, SiO_2_ nanoparticles, and nanopores. As seen from the magnified SEM images, the unordered nano-scaled ATP did not influence the formation of the porous structure of the ATP/SiO_2_ aerogels. Additionally, there were some large pores in the HAS-F and densification phenomenon in the HAS-A, which is detrimental to the specific surface areas. By contrast, the AS-S and HAS-S samples exhibited significant homogeneous pore structures. Furthermore, the TEM images (Figure 4f) show that the as-prepared ATP/SiO_2_ aerogels using different drying methods displayed random interconnected networks of nanometer-sized SiO_2_ aerogel particles and club-shaped ATP, with interconnected amorphous silica particles surrounding the ATP. Meanwhile, the ATP could form hydrogen bonds with silica particles during the reaction, which could strengthen the crosslinked structures of the ATP/SiO_2_ aerogels and resist the greater capillary pressure during the drying process. Satisfactorily, compared to other aerogel composites strengthened by coarse fibers, the nanoscale club-shaped ATP was conducive to improving the mechanical performance of the prepared spherical ATP/SiO_2_ aerogels without destroying the internal pore structures [19]. The EDS spectrums of AS-S and HAS-S are presented in Figure 5, and the concentration of C, O, and Si elements are presented in Table 2. The apparent concentration of the C element increased from 23.8 wt% (AS-S) to 40.1 wt% (HAS-S). In addition, the homogenous distribution of the C element of HAS-S further proves the successful modification of MTMS.

**Figure 6:**
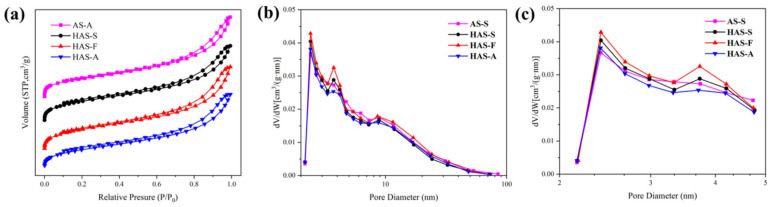
(**a**) The N_2_ adsorption–desorption curve and (**b**,**c**) BJH pore size distribution curve of AS-S, HAS-S, HAS-F, and HAS-A.

The N_2_ adsorption–desorption isotherms and BJH pore size distribution of the spherical ATP/SiO_2_ aerogels are described in Figure 6. All isotherms are Type IV based on the IUPAC classification, reflecting the characteristic of mesoporous materials. The adsorption–desorption curves form closed hysteresis loop, which is generally caused by the capillary condensation in the mesopores. The rapid adsorption process in the low-pressure region (0–0.1) is caused by the micropores inside the aerogel matrix. It is shown in Table 1 that the specific surface area of the as-prepared ATP/SiO_2_ aerogels (with the content of ATP over 60 wt%) was much higher than that of pure ATP, resulting from the improvement in the network skeleton structures of the ATP/SiO_2_ aerogel composites. Additionally, the specific surface areas of the aerogels obtained by different treatment techniques showed a decreasing trend (S_SCD_ > S_FD_ > S_APD_), corresponding to the values of 248.7–267.4 m^2^/g, 241.7 m^2^/g, and 218.5 m^2^/g, respectively. The HAS-A showed the minimum specific surface area because of the particle agglomeration and closed pores appearing during the APD process (shown in Figure 4d). The HAS-S had higher specific surface areas than AS-S, which can be explained by the fact that the grafting of MTMS improves the hydrophobic property of ATP/SiO_2_ aerogel without damaging the nano-porous microstructure. Comparing AS-S to HAS-S, the average pore size and pore volume decreased from 8.3 nm and 0.41 cm^3^/g to 7.9 nm and 0.37 cm^3^/g, respectively. This is because some organic groups enter the larger pores of spherical ATP/SiO_2_ aerogels with the grafting of the CH_3_-(SiOH)_3_ group, which is favorable to increasing the porosity of HAS-S [41]. Furthermore, owing to the high capillary pressure of the solvent evaporation and shrinkage of the pore structure in the APD process, the pore volume of the HAS-A decreased significantly in comparison with the other hydrophobic samples. Compared with HAS-S, HAS-F exhibited lower specific surface area and higher pore volume due to the destruction of the original pore structure during the crystallization and sublimation of the solvent. As revealed in Figure 6b, all spherical ATP/SiO_2_ aerogels showed a broad pore size distribution in the range of 2–50 nm. After the hydrophobic modification, the peak of the micropores slightly shifted to higher values (Figure 6c), which can be explained by the fact that the pores in the aerogel are filled with the hydrolysis products of MTMS [41]. This indicates that the hydrophobic modification was beneficial to the microporous structure of the aerogels. Therefore, all spherical ATP/SiO_2_ aerogels had a good specific surface area and porous network structure, revealing the feasibility of the multipath preparation via the SCD, FD, and APD techniques.

**Figure 7:**
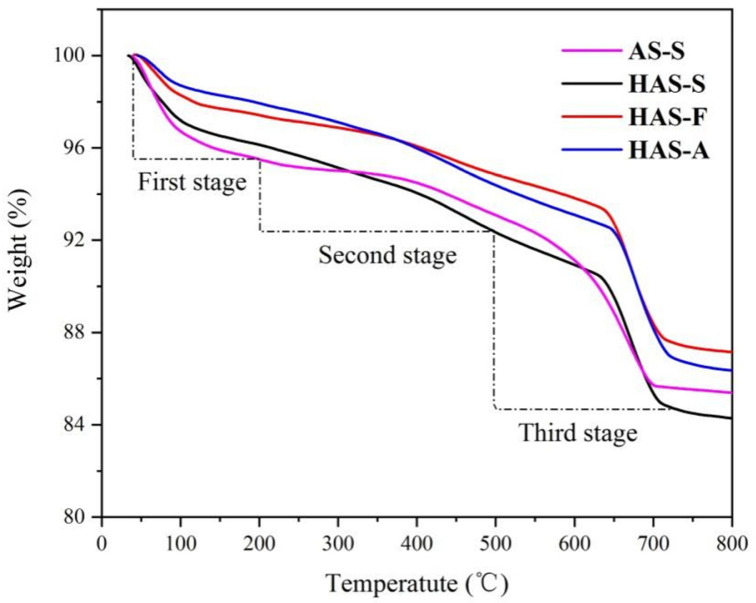
TG curves of AS-S, HAS-S, HAS-F, and HAS-A.

The TG curves of the prepared spherical ATP/SiO_2_ aerogels are displayed in Figure 7, under flowing air. The weight loss rates of the AS-S, HAS-S, HAS-F, and HAS-A were 14.62%, 15.72%, 12.85%, and 13.67%, respectively. The weight loss stage was mainly divided into three parts. The first stage, consisting of the volatilization of the residual solvent and the adsorbed water, appeared at 50–200 °C. In this stage, the weight loss rate of the hydrophobic samples was lower than AS-S (4.51%), which reflects the hydrophobicity of the MTMS modification. Considering the residual solvent, water molecules, and CO_2_ during the SCD process, the weight loss rates of AS-S (4.51%) and HAS-S (3.87%) were significantly higher than those of HAS-F (2.59%) and HAS-A (2.17%). In the second stage between 200 °C and 500 °C, the weight loss was assigned to the elimination of the crystal water in the ATP and the condensation of Si-OH. The weight loss in the third stage (over 500 °C) was mainly attributed to the oxidative decomposition of Si-CH_3_ and the removal of structural water in the ATP [27]. It is noted that a characteristic temperature point appeared in the hydrophobic sample at approximately 630 °C, which implies the complete decomposition of the Si-CH_3_ group [42].

### 3.3. Effect of Heat Treatment on Physical Characteristic and Morphological Properties of HAS-A

**Figure 8:**
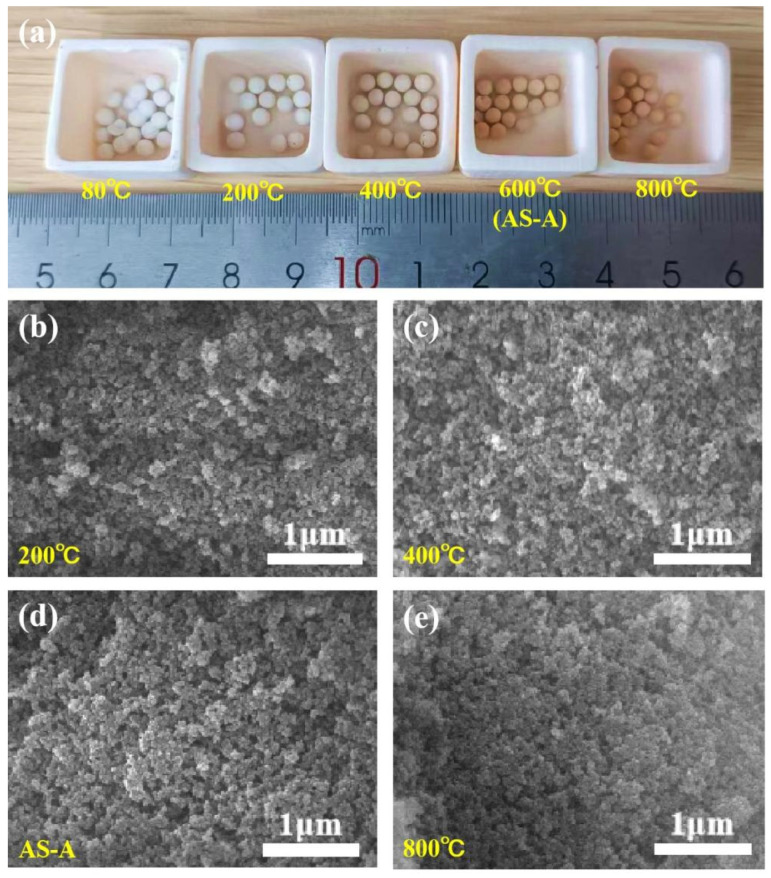
(**a**) Photographs and (**b**–**e**) SEM images of the HAS-A with different heat treatment temperatures.

**Figure 9:**
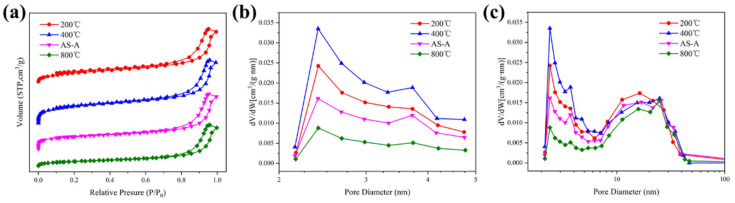
(**a**) N_2_ adsorption–desorption and (**b**,**c**) BJH pore size distribution curve of the HAS-A at different calcination temperatures.

The influence of the heat treatment temperatures on the morphology, microstructure, and pore structure is shown in Figure 8 and Figure 9. Heat treatment is a simple and efficient way to modify the crosslinking structure and chemistry of aerogels [43]. The 600 °C heat-treated HAS-A is denoted as AS-A. As the calcination temperature increased, the appearance of the color of the HAS-A gradually changed from off-white to brick red (Figure 8a), which is mainly caused by the component of ATP. It is found from Figure 8b–e that the HAS-A heat treated at 400 °C showed a highly homogeneous porous structure. Due to the pyrolysis of -CH_3_ and condensation among -OH, there was a large amount of silica particles aggregated inside the aerogel, and the uniform pore structure was damaged after calcination at 600 °C. A denser silica structure of the as-prepared HAS-A heat treated at 800 °C was clearly observed. All the curves in Figure 9a are type IV isotherms, suggesting that mesopores are still present in the aerogel despite the high temperature heat treatment. Consequentially, the pore size distributions of the heat-treated samples are shown in Figure 9b,c. From 200 °C to 400 °C, the peak of the micropores increased and then decreased after 600 °C. This is because at 200–400 °C, the excess organic matter inside the pores of the HAS-A decomposed, resulting in extra pores. At 600–800 °C, the aerogel particles inside the HAS-A will be aggregated and block the pore channels [44]. The pore structure of the HAS-A at different calcination temperatures is shown in Table 3. The specific surface area increased dramatically from 231.5 m^2^/g to 337.7 m^2^/g, with the treatment temperature increasing from 200 °C to 400 °C. Simultaneously, it was observed that the total pore volume changed from 0.40 cm^3^/g to 0.43 cm^3^/g, while the average pore size changed from 11.3 nm to 10.6 nm. However, owing to the oxidation of -CH_3_ groups and condensation between -OH, a further increase in the calcination temperature was not favorable for the specific surface area and total pore volume.

**Figure 10:**
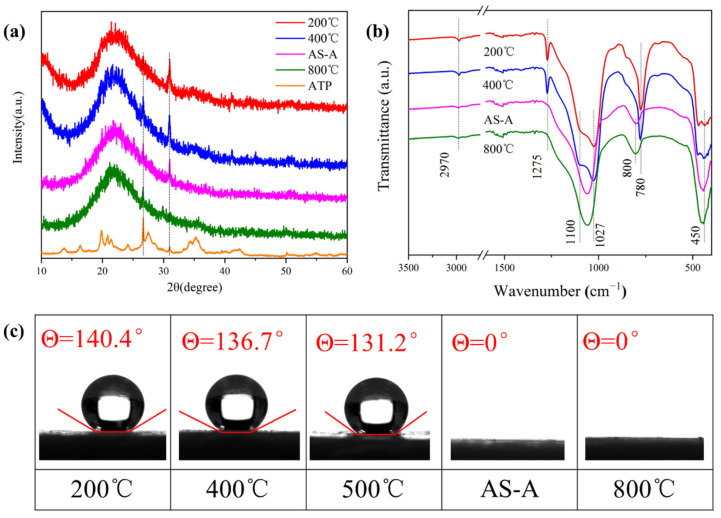
(**a**) The XRD patterns, (**b**) FT-IR spectra, and (**c**) water contact angles of the HAS-A with different heat treatment temperatures.

Figure 10a shows the XRD pattern of the heat-treated HAS-A. The characteristic peaks at 2θ = 27.6° and 30.9° were assigned to the (240) and (400) crystal planes of the ATP [27]. After a 600 °C calcination, the characteristic peak at 30.9° decreased significantly, indicating that the crystal structure of the ATP had been disrupted. After a 800 °C calcination, the characteristic peak at 30.9° gradually disappeared, indicating the complete destruction of the ATP crystal structure. A broad peak at 2θ = 21.5° was clearly identified in all samples, indicating that the silica aerogels preserve their original amorphous structure despite high temperature calcination [44]. The FT-IR spectra of the HAS-A heat treated at different temperatures are depicted in Figure 10b. The -CH_3_ groups are observed at the absorption peaks of 780 cm^−1^, 1275 cm^−1^, and 2970 cm^−1^. Those bands of the −CH_3_ groups disappeared after a 600 °C calcination, indicating the pyrolysis of -CH_3_ [42]. Moreover, Figure 10c shows the water contact angles of the HAS-A treated at different temperatures. The hydrophobicity of the HAS-A remained stable at 500 °C (200 °C, 400 °C, and 500 °C, corresponding to the values of 140.4°, 136.7°, and 131.2°, respectively), while the HAS-A converted to hydrophilic AS-A completely at 600 °C, with a water contact angle value of 0°, which is in accordance with the representation results in the TG (Figure 7) and FT-IR (Figure 10b). The transition from hydrophobic to hydrophilic in the spherical ATP/SiO_2_ aerogels is conducive to the adsorption of soluble dyes in aqueous solutions. Therefore, the AS-A were used as adsorbents for studying the adsorption performance.

### 3.4. Adsorption Studies

**Figure 11:**
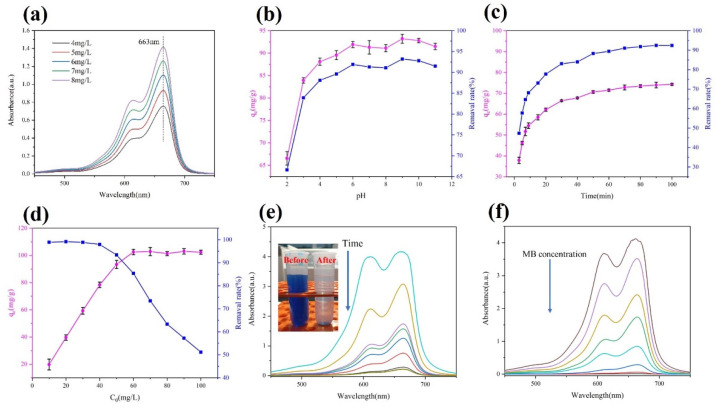
(**a**) Absorbance curves of the MB at different concentrations; (**b**–**d**) adsorption efficiency of the AS-A at different pH, time, and initial concentration values on the MB; (**e**,**f**) absorption spectra with the time and initial concentration of the MB.

To observe a visible change in the MB [7], Figure 11a exhibits the absorbance curves of MB at different concentrations. The adsorption capacity of the AS-A at different pH values, times, and MB concentrations is discussed as follows.

#### 3.4.1. Effect of the Solution pH on MB Removal by AS-A

In this experiment, the pH was adapted from 2 to 11, and 50 mg of AS-A was used to adsorb MB at 50 mg/L for 100 min. The result in Figure 11b shows that the removal efficiency improved with the increase in the value of pH. The MB preferred to adsorb on AS-A under alkaline conditions, and the adsorption efficiency could reach 93.2% at a pH value of nine. Under acidic conditions, the adsorption capacity of AS-A is limited. This is due to the presence of excess hydrogen ions under acidic conditions, which leads to the protonation of functional groups on the AS-A surface during the adsorption process. Under alkaline conditions, AS-A has more adsorption sites on its surface, and the adsorption efficiency of MB will increase. Consequently, the pH value for the subsequent experiments was set to nine.

#### 3.4.2. Effect of Adsorption Time on MB Removal

In this part, the adsorption quantities from 0 to 100 min were studied to explore the adsorption equilibrium time. The pH value and MB concentration were set to 9 and 40 mg/L, respectively. As shown in Figure 11c, the adsorption capacity increased rapidly to 69.2% within 10 min. Figure 11e presents the spectra of the absorbance of MB with time. After 90 min, the adsorption equilibrium was achieved. The corresponding adsorption efficiency and capacity of the MB were 92.45% and 73.96 mg/g, respectively. After another 10 min, there was no noticeable change in the adsorption rate. Therefore, in subsequent experiments, the adsorption time was set to 100 min.

#### 3.4.3. Effect of Initial Concentration on MB Removal

The effect of the initial MB concentration (10–100 mg/L) on the adsorption efficiency is shown in Figure 11d. Figure 11f shows the variation in the absorption spectra for different initial concentrations of MB. The adsorption capacity increased and then gradually stabilized with the increasing MB concentration. When the MB concentration reached 60 mg/L, the adsorption capacity reached 102.50 mg/g. As the concentration of MB was further increased, there were no significant changes in the adsorption capacity. This indicates that 50 mg of AS-A could not provide enough adsorption sites to remove the excess MB from the solution. 

#### 3.4.4. Adsorption Kinetics

The adsorption kinetics is closely related to the contact time, which can reflect the adsorption mechanism of the absorbent. Kinetic models were used to evaluate the experimental data:

Pseudo-first-order equation:

$$\ln\left(q_{e}-q_{t}\right)=\ln q_{e}-k_{1}t$$

Pseudo-second-order equation:

$$\frac{t}{q_{t}}=\frac{1}{k_{2}q_{e}^{2}}+\frac{1}{q_{e}}t$$

where *k*_1_ and *k*_2_ (g/(mg·min)) represent the pseudo-first-order and pseudo-second-order rate constants, respectively; *q_e_* (mg/g) is the equilibrium adsorption quantity; *q_t_* (mg/g) is the quantity adsorbed at different times (“*t*”).

**Figure 12:**
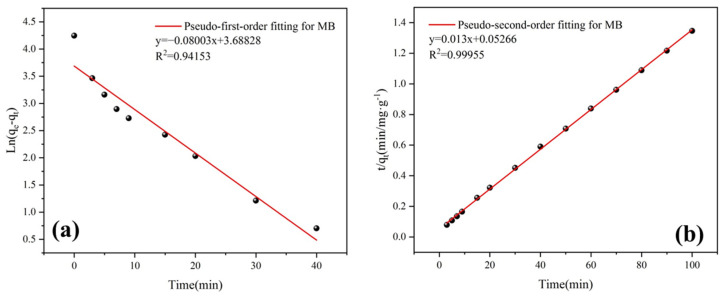
(**a**) Pseudo-first-order equation; (**b**) pseudo-second-order equation.

The model fitting results and the calculated kinetic parameters are shown in Figure 12 and Table 4. Compared with the pseudo-first-order, the experimental data confirm that the adsorption process was consistent with the pseudo-second-order model with high R^2^ values of 0.99955. This suggests that the adsorption efficiency is highly dependent on the quantity of unoccupied active sites [45]. The *q_e_* values of the pseudo-second-order model (76.90 mg/g) were found to be essentially the same as the experimental results (73.96 mg/g), indicating that the pseudo-second-order kinetic model can be employed for the adsorption of MB on AS-A.

#### 3.4.5. Adsorption Isotherms

The equilibrium adsorption isotherms play an important role in the interaction between the adsorbent and the adsorbate, which can explore the maximum adsorption capacity of the adsorbent. The Langmuir equation and the Freundlich equation are shown as follows:

Langmuir equation:

$$\frac{C_{e}}{q_{e}}=\frac{1}{q_{max}}C_{e}+\frac{1}{k_{L}q_{max}}$$

**Figure 13:**
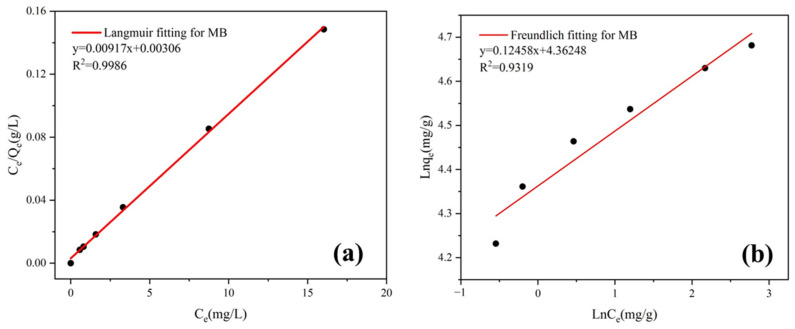
(**a**) Langmuir isotherm model; (**b**) Freundlich isotherm model.

Freundlich equation:

(3) $$\ln q_{e}=\ln k_{F}+\frac{1}{n}\ln C_{e}$$

where *q_e_* (mg/g) is the adsorption amount at adsorption equilibrium; *q_max_* (mg/g) stands for the maximum adsorption capacity; *C_e_* (mg/L) represents the equilibrium concentration; *k_L_* and *k_F_* are the constants of the Langmuir isotherm equation and Freundlich isotherm equation; and 1/n is the adsorption intensity parameter. The fitting results of the Langmuir and Freundlich equations are shown in Figure 13 and Table 5.

The Langmuir model fits the experimental data best, with R^2^ values of 0.9986, which proves that the sorption process of MB on AS-A belongs to monolayer adsorption [45]. The maximum adsorption capacity derived from the Langmuir adsorption model was 109.05 mg/g, which closely corresponds to the experimental data (102.50 mg/g). The adsorption performance of the AS-A was compared with the other reported adsorbents (Table 6). The AS-A obtained by heat treatment at 600 °C had excellent adsorption performance on MB in comparison with the other adsorbents. Although it has no advantage in terms of specific surface area, the combination of attapulgite and silica aerogel greatly improved the adsorption of the composite. This reveals that AS-A is a potential adsorbent for dye wastewater.

#### 3.4.6. Recycling Studies

**Figure 14:**
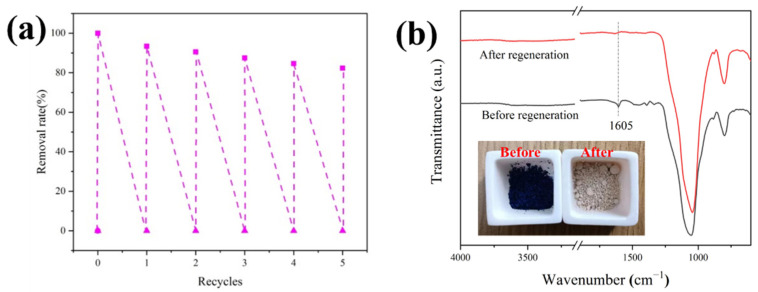
(**a**) Removal rate of AS-A during five cycles (C_0_ = 50 mg/L, V = 100 mL, m = 50 mg, pH = 9, and t = 100 min); (**b**) FT-IR spectra of AS-A before and after regeneration.

The regenerative adsorption of the adsorbent is a vital factor in measuring its suitability in practical applications. In the recycling test, AS-A was heat treated at 600 °C for 3 h in a muffle furnace to eliminate the adsorbed dye. As shown in Figure 14a, the adsorption capacity of AS-A remained at 82.32% after five adsorption cycles, indicating that the adsorption of MB on AS-A was stable for multiple cycles. After the regeneration, the disappearance of MB aromatic group at 1603 cm^−1^ illustrates that MB can be completely removed by calcination at 600 °C (Figure 14b) [52].

#### 3.4.7. Adsorption Mechanism

**Figure 15:**
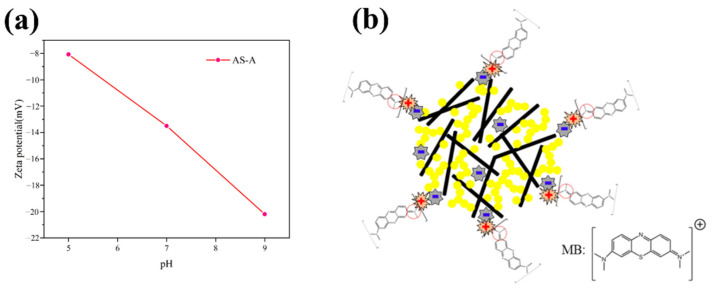
(**a**) Zeta potential of AS-A; (**b**) mechanisms of MB adsorption on AS-A.

The zeta potential of the AS-A was negative in the pH range of 5–9 (Figure 15a), indicating that the surface of the AS-A exhibited negative charges. In addition, the amount of negative charge on the AS-A surface tended to increase with the increase in the pH. As shown in Figure 15b, MB is a typical cationic dye with a positive charge [53]. The negatively charged AS-A had a significant adsorption effect on the positively charged MB, resulting in electrostatic attraction between MB and AS-A. Therefore, the adsorption mechanism of AS-A on MB is mainly an electrostatic effect. The surface negative charge of AS-A has great attraction to MB with positive charge, which leads to an excellent adsorption performance on MB. 

## 4. Conclusions

In this work, the spherical ATP/SiO_2_ aerogels were successfully synthesized by three different drying techniques (SCD, FD, and APD). The spherical ATP/SiO_2_ aerogels dried with three techniques possessed a complete macromorphology and homogeneous porous network structures. The control of the size of the obtained spherical particles will be included in the next step of the study. The club-shaped ATP was well dispersed in the aerogel matrix without agglomeration, and the silica particles were closely attached to the surface of the ATP. The average pore diameter and specific surface area of the spherical ATP/SiO_2_ aerogels prepared by three drying techniques were in the range of 6.8–8.6 nm and 218.5–267.4 m^2^/g, respectively. The BET specific surface area of the HAS-A after the 400 °C heat treatment sharply increased to 333.7 m^2^/g. Compared to the other adsorbents, AS-A showed a higher adsorption capacity of 102.50 mg/g for MB. After five cycles of regeneration, the adsorption efficiency of AS-A could still maintain 82.32%, exhibiting a good reusability. Therefore, these porous spherical ATP/SiO_2_ aerogels with multipath preparation and excellent adsorption performance are expected to have applications in dye wastewater treatment.

## Figures and Tables

**Table 1:** The physical parameters of the ATP and spherical ATP/SiO_2_ aerogels prepared by the different drying methods.

Sample	ρ (g/cm^3^)	S_BET_ (m^2^/g)	Pore Size (nm)	Pore Volume (cm^3^/g)
ATP	2.41	160.5	8.6	0.27
AS-S	0.46	248.7	8.3	0.41
HAS-S	0.53	267.4	7.9	0.37
HAS-F	0.56	241.7	8.1	0.35
HAS-A	0.65	218.5	6.8	0.33

**Table 2:** The C, O, Si, and metal element (Mg, Al, K, Ca, Fe) concentration of AS-S and HAS-S.

Sample	C (wt%)	O (wt%)	Si (wt%)	Metal (wt%)
AS-S	23.8	42.9	24.6	8.7
HAS-S	40.1	40.8	17.2	2.3

**Table 3:** Pore structure of the HAS-A after calcination.

Sample	S_BET_ (m^2^/g)	Pore Size (nm)	Pore Volume (cm^3^/g)
200 °C	231.5	11.3	0.40
400 °C	337.7	10.6	0.43
AS-A	246.6	12.4	0.40
800 °C	107.1	13.9	0.33

**Table 4:** Kinetic parameters of MB adsorption on AS-A.

Pseudo-First-Order Model			Pseudo-Second-Order Mode		
*q_e_* (mg/g)	*k*_1_	R^2^	*q_e_* (mg/g)	*k*_2_	R^2^
39.98	0.08003	0.94153	76.90	0.00321	0.99955

**Table 5:** Constants for the adsorption isotherms of MB on AS-A.

Langmuir Equation			Freundlich Equation		
*q_max_* (mg/g)	*k_L_*	R^2^	*k_F_*	1/*n*	R^2^
109.05	2.997	0.9986	78.45	0.12458	0.9319

**Table 6:** Comparison of MB adsorption capacities with different adsorbents.

Adsorbent	Treatment Method	S_BET_ (m^2^/g)	*q_max_* (mg/g)	Reference
SiO_2_(AG)	Heat treated at 500 °C for 3 h	902.0	9.53	Yi et al. 2019 [10]
HSA	Heat treated at 600 °C for 3 h	888.7	51.16	Wei et al. 2018 [11]
SAs	Mg^2+^ soaked	468.2	40.40	Yang et al. 2020 [12]
Attapulgite	Heat treated at 700 °C for 4 h	-	78.11	Chen et al. 2011 [27]
Ag/SiO_2_	Ag decorated	208.0	55.00	Hu et al. 2019 [46]
MPCMs	Heat treated at 700 °C for 30 min	480.3	56.44	Zhang et al. 2021 [47]
ZIF-8	Fe decorated	329.9	9.09	Mahmoodi et al. 2021 [48]
CaCO**_3_**@STA/PAM/TOCN	-	39.2	101.01	Li et al. 2022 [49]
CCGA	-	225.2	96.10	Shimizu et al. 2022 [50]
CNCS/SiO**_2_** aerogel	-	440.7	190.85	Ruan et al. 2022 [51]
AS-A	Heat treated at 600 °C for 3 h	246.6	102.50	This work

## Data Availability

The data that support the findings of this study are available from the corresponding author upon reasonable request.
